# Supplementary material for: Management of Out-of-Hospital Cardiac Arrest during COVID-19: A Tale of Two Cities
Source: J Clin Med. 2022 Sep 1;11(17):5177. doi: 10.3390/jcm11175177 (PMC9457434; doi:10.3390/jcm11175177)
Supplement: Supplementary file 1 [file jcm-11-05177-s001.zip › jcm-1872932-supplementary.pdf]

Journal of
Clinical Medicine

SUPPLEMENTAL DATA

(a) Singapore

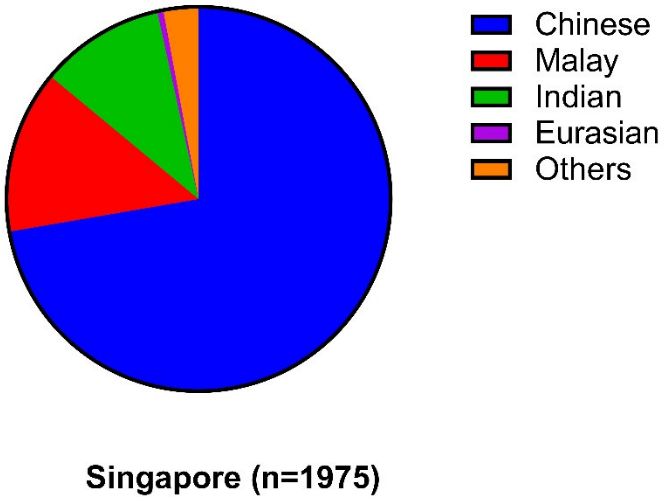

(b) Atlanta

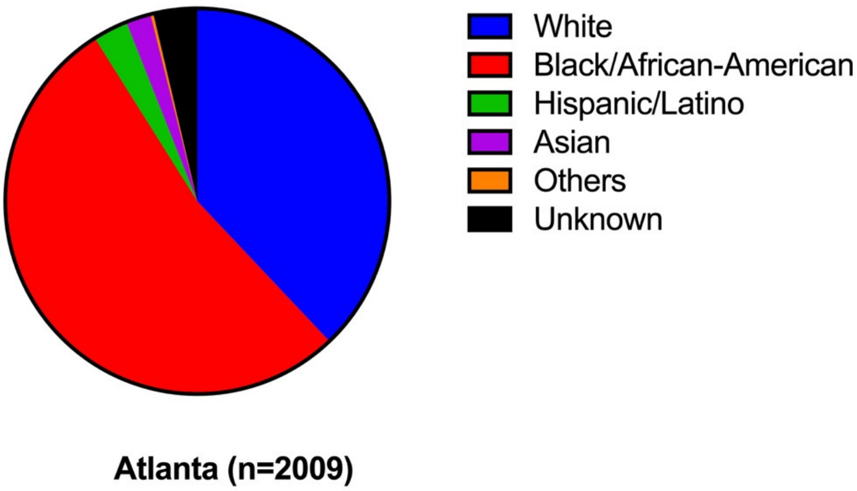

Supplemental Figure S1. Racial distribution of adult, EMS-treated OHCA. Abbreviations: EMS, Emergency Medical Services; OHCA, out-of-hospital cardiac arrest.

Supplemental Table S1. Characteristics of EMS-treated OHCA and outcome, by city, location of arrest and period.

(a): Singapore				
	Singapore			
	Home/Residential		Public Areas	
	Pandemic N=808	Pre-Pandemic N=724	Pandemic N=105	Pre-Pandemic N=155
**Demographics**				
Age in years, median [Q1, Q3]	75.0 [63.0, 84.0]	73.0 [62.0, 84.0]	56.0 [50.0, 64.0]	59.0 [50.0, 69.0]
Male gender, n (%)	489 (60.5%)	420 (58.0%)	98 (93.3%)	131 (84.0%)
**Event information, n (%)**				
Presumed cardiac aetiology	735 (91.0%)	638 (88.1%)	98 (93.3%)	145 (93.5%)
Initial shockable rhythm	106 (13.1%)	87 (12.0%)	39 (37.1%)	59 (38.1%)
Witnessed arrest				
- Unwitnessed	345 (42.7%)	424 (58.6%)	30 (28.6%)	58 (37.4%)
- Bystander witnessed	384 (47.5%)	229 (31.6%)	59 (56.2%)	84 (54.2%)
- EMS witnessed	79 (9.8%)	71 (9.8%)	16 (15.2%)	13 (8.4%)
**Pre-hospital resuscitation, n (%)**				
Bystander CPR	458(62.8%)	440 (67.4%)	53 (59.6%)	98 (69.0%)
Pre-hospital defibrillation	152 (18.8%)	127 (17.5%)	52 (49.5%)	90 (58.1%)
**EMS response times in min, median [Q1, Q3]***				
EMS response time	8.62 [6.91, 10.4]	7.93 [6.55, 9.59]	9.28 [7.30, 11.2]	8.87 [6.66, 11.5]
Total response time	12.9 [10.8, 15.2]	11.1 [9.38, 13.2]	12.2 [10.2, 15.7]	12.3 [9.53, 15.2]
- Call received to dispatch	1.97 [1.43, 2.72]	2.08 [1.57, 2.70]	2.33 [1.77, 3.48]	2.32 [1.68, 3.31]
- Dispatch to scene arrival	6.37 [4.93, 8.10]	5.87 [4.58, 7.48]	6.43 [4.97, 8.95]	6.50 [4.46, 9.03]
	4.03 [2.85, 5.57]	2.93 [1.83, 4.00]	3.33 [1.32, 5.33]	2.17 [0.992, 4.22]

- Scene arrival to patient's side<br>Time at scene	25.3 [21.9, 29.0]	23.7 [20.8, 27.2]	22.5 [17.6, 26.2]	20.3 [16.6, 23.6]
**Patient outcomes, n(%)****				
Transported	713 (88.2%)	670 (92.3%)	104 (99.0%)	155 (100%)
Survived to hospital admission	92 (11.4%)	96 (13.3%)	24 (22.9%)	57 (36.8%)
Survived to hospital discharge	21 (2.6%)	27 (3.7%)	13 (12.4%)	29 (18.7%)
Discharged with good neurological outcome	16 (2.0%)	22 (3.0%)	12 (11.4%)	29 (18.7%)

(b): Atlanta

	Atlanta			
	Home/Residential		Public Areas	
	Pandemic<br>N=792	Pre-Pandemic<br>N=620	Pandemic<br>N=90	Pre-Pandemic<br>N=138
**Demographics**				
Age in years, median [Q1,Q3]	65.0 [53.0, 76.0]	65.5 [53.0, 70.0]	52.5 [45.0, 63.0]	60.0 [44.0, 69.0]
Male gender, n(%)	413 (52.1%)	345 (55.6%)	74 (82.2%)	104 (75.4%)
**Event information, n(%)**				
Presumed cardiac aetiology	668 (84.3%)	540 (87.1%)	75 (83.3%)	124 (89.9%)
Initial shockable rhythm	114 (14.4%)	104 (16.8%)	34 (37.8%)	52 (37.7%)
Witnessed arrest				
- Unwitnessed	372 (47.0%)	290 (46.8%)	31 (34.4%)	42 (30.4%)
- Bystander witnessed	293 (37.0%)	231 (37.3%)	45 (50.0%)	81 (58.7%)
- EMS witnessed	127 (16.0%)	99 (16.0%)	14 (15.6%)	15 (10.9%)

**Pre-hospital resuscitation, n(%)**				
Bystander CPR	280 (35.4%)	196 (37.6%)	29 (32.2%)	69 (56.1%)
Pre-hospital defibrillation	196 (24.7%)	162 (26.1%)	47 (52.2%)	68 (49.3%)
**EMS response times in min, median [Q1,Q3]***				
EMS response time	9.77 [6.93, 12.7]	9.15 [6.68,12.2]	8.19 [6.01, 10.9]	8.52 [6.00, 12.0]
Total response time	11.5 [9.00, 15.0]	11.0 [8.39, 14.0]	9.94 [7.68, 14.2]	10.0 [6.75, 13.8]
- Call received to dispatch	0.583 [0.125, 1.13]	0.633 [0.100, 1.47]	0.633 [0.200, 1.17]	0.850 [0.296, 1.95]
- Dispatch to scene arrival	8.48 [6.00, 11.4]	8.00 [5.72, 11.0]	7.18 [5.00, 9.19]	7.00 [4.20, 9.55]
- Scene arrival to patient's side	1.53 [0.950, 2.77]	1.22 [0.817, 2.02]	1.23 [0.929, 2.03]	1.00 [0.517, 1.91]
Time at scene	23.2 [18.0, 31.3]	20.5 [15.3, 27.2]	20.5 [14.0, 27.0]	18.5 [12.9, 23.1]
**Patient outcomes, n (%)****				
Transported	601 (75.9%)	552 (89.0%)	89 (98.9%)	134 (97.1%)
Survived to hospital admission	138 (17.6%)	136 (21.9%)	37 (43.0%)	47 (34.1%)
Survived to hospital discharge	47 (6.01%)	46 (7.4%)	20 (23.3%)	22 (15.9%)
Discharged with good neurological outcome	31 (4.0%)	34 (5.5%)	16 (18.6%)	18 (13.0%)

Numbers are n (%) for categorical variables and median (Q1-Q3) for continuous variables.

*Data from Atlanta are not available for: 660 (40.2%) call received to dispatch; 385 (23.5%) dispatch to scene arrival; 668 (40.7%) scene arrival to patient's side; 655 (39.9%) EMS response time; 658 (40.1%) total response time; 573 (34.9%) scene time.

*Data from Singapore are not available for: 153 (8.5%) scene time.

**Data for survival to hospital discharge are not available for 1 patient from Singapore and 14 patients from Atlanta.

Abbreviations: EMS, emergency medical services; OHCA, out-of-hospital cardiac arrest; CPR, cardiopulmonary resuscitation
